# Effects of Alkaline Cleaning Agents on the Long-Term Performance and Aging of Polyethersulfone Ultrafiltration Membranes Applied for Treatment of Car Wash Wastewater

**DOI:** 10.3390/membranes14060122

**Published:** 2024-05-24

**Authors:** Marek Gryta, Piotr Woźniak, Sylwia Mozia

**Affiliations:** Faculty of Chemical Technology and Engineering, West Pomeranian University of Technology in Szczecin, ul. Pułaskiego 10, 70-322 Szczecin, Poland; piotr.wozniak@zut.edu.pl (P.W.); sylwia.mozia@zut.edu.pl (S.M.)

**Keywords:** car wash wastewater, ultrafiltration, PES, fouling, alkaline cleaning agents, membrane degradation

## Abstract

The commercial ultrafiltration polyethersulfone (PES) membranes (10 and 100 kDa) blended with polyvinylpyrrolidone (PVP) were applied for the filtration of car wash wastewater. Periodical membrane rinsing with water did not prevent fouling and a decrease in permeate flux was observed. Fouling was reduced by washing the membranes with cleaning agents, which are used in car washes to clean wheels and remove insects. In addition to surfactants, these agents contain NaOH, hence the pH value of cleaning solutions was over 11. Long-term contact with such solutions resulted in the removal of PVP from the membrane matrix and an increase in pore size. The PES membranes were soaked in an alkaline solution (pH = 11.5) for 20 months, after which the 200 kDa dextran rejection decreased from 95% to 80%. To compare with the static degradation conditions, 8 weeks of alkaline agent filtration was realized, after which the dextran (200 kDa) rejection decreased below 50%. This indicated that the cross-flow of alkaline agents can accelerate the removal of components building the membrane matrix. Despite membrane degradation, the separation efficiency (the rejection of chemical oxygen demand—COD, turbidity, and surfactants) during the treatment of synthetic car wash wastewater was similar to that obtained for pristine membranes.

## 1. Introduction

Wastewater generated during car washing contains many substances hazardous to the environment, such as mineral oil, wax, and surfactants, and therefore requires treatment [1,2]. The processes used for this purpose typically include conventional methods based on coagulation, flocculation, sedimentation, and sand filtration [1,2,3,4,5]. In order to reduce costs, the utilization of natural plant coagulants is proposed, which also facilitates waste disposal [2]. The use of the integrated system to treat car wash wastewater is effective for large automatic car washes [6]. Designs for underground and above-the-ground-level installations were presented in previous work [7]. However, such large installations are not accepted by the owners of small manual car washes who want cheap and simple cleaning installations.

More than 100–200 L of water is used to wash a car, which gives an annual worldwide consumption of millions of cubic meters [5,8]. For this reason, in some countries, legal regulations require that 40–80% of the water should be recovered for washing cars [9,10]. Such possibilities are created by membrane processes [8,11,12], which also allow the construction of small installations that can be used in manual car washes. Car washing is a multi-stage process, most often ending with rinsing the car with reverse osmosis permeate. For the initial stages, the water obtained from wastewater separated by ultrafiltration (UF) can be used as processing water [8,13].

A phenomenon that limits the use of membrane separation is fouling [14,15]. Contaminants present in car wash wastewaters, such as suspensions, oils, and fats, form deposits on the surface of the membranes, which are difficult to remove by washing modules only with water and surfactant solutions [13]. A much better result is achieved by using chemical cleaning [16]. In this case, solutions containing acids, NaOH, or NaClO are used to wash the membrane [17]. Such solutions, especially those containing NaClO, are aggressive, thus periodically repeated washing may cause the degradation of the membranes [18,19,20]. The feasibility of implementing the process largely depends on the membrane durability. For these reasons, when selecting membrane modules for the UF process, preliminary tests should be performed on their resistance to solutions used in a given car wash. As a rule, membrane aging is tested by soaking them in cleaning solutions for several months [21,22]. However, such static measurements may not fully reflect the dynamic conditions in cross-flow modules. Thus, in the presented work, the filtration of a solution containing surfactants and NaOH (pH = 11.5) was carried out.

Polymeric membranes with high chemical resistance are typically made of hydrophobic polymers such as polyvinylidene fluoride (PVDF) or polytetrafluoroethylene (PTFE). However, these are expensive materials, so membrane modules made of less resistant but much cheaper polymers are more commonly used [15]. In many applications, polyethersulfone (PES) membranes, which can operate in a wide pH range from 2 to over 12, demonstrated good performance [15,17,23,24]. Membranes made of this polymer have been successfully used to treat wastewater generated during car washing [13,25]. The components of this wastewater caused significant fouling, therefore, periodical cleaning of the PES membranes was necessary. Chemical cleaning usually removes fouling and restores primary membrane performance.

The good resistance of several types of PES membranes to NaOH and HNO_3_ solutions was demonstrated in tests lasting 180 days [21]. However, the hydrophobic nature of PES makes these membranes susceptible to fouling [24,26]. In order to reduce it, PES membranes often contain hydrophilic additives such as polyvinylpyrrolidone (PVP) [18,27]. Its addition increases the hydrophilicity of the membrane surface and, as a result, adsorptive fouling is much lower [28]. Unfortunately, PVP is not resistant to the action of alkaline cleaning solutions [29,30]. NaOH solutions caused its degradation, which resulted in the leaching of PVP from the PES membranes [31]. This should be attributed to the NaOH-catalyzed hydrolysis, which led to the breakage of some polymer chains [32,33]. These degradation processes occurred mainly during the initial 2–3 months of the contact of the polymer with alkaline solutions [22,34].

Cleaning agents designed for membranes, such as P3 Ultrasil 11 recommended for UF membranes [35,36], may cause damage to car paint. For this reason, car wash owners are against using additional chemicals in the car washing installation. Therefore, a concept was created to clean the membranes with agents used for washing cars [13,25]. The results of previous work have shown that alkaline agents used to clean wheels and remove insects can be applied for this purpose [13]. These cleaning agents, similarly to P3 Ultrasil 11, contain NaOH, EDTA tetrasodium salt, and surfactants [13,35,37]. By washing PES membranes every 5–7 h for 30 min with solutions (0.3–0.5%) of these agents, a stable permeate flux at a level of 70–80% of the initial flux was obtained during 100 h of wastewater filtration tests [13]. However, this result was obtained from a test conducted for only a few days. Taking into account that the negative impact of NaOH solutions may become apparent after repeated chemical cleaning operations, for the commercialization of the technology much larger testing periods are required [30]. For this reason, in the presented work, several months of UF car wash wastewater tests were carried out with cyclical washing of membranes with alkaline cleaning agents. To enhance the effect of PES degradation, in the experiments described here membranes soaked for 20 months in alkaline solutions were applied. To test the resistance of the membranes to cross-flow conditions, several weeks of the filtration of the tested alkaline agents were also carried out.

## 2. Materials and Methods

### 2.1. UF Installation

**Figure 1:**
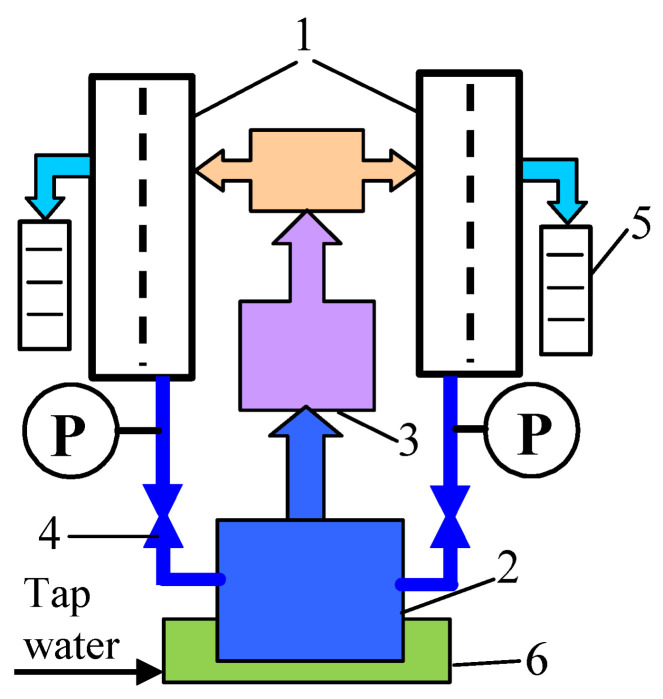
UF installation with two plate-and-frame modules. Here, 1—membrane module, 2—feed tank, 3—pump, 4—needle valve, 5—measurement cylinder, 6—water bath, and P—manometer.

Studies on the degradation of membranes under dynamic conditions have been carried out in a laboratory UF installation, the diagram of which is shown in Figure 1. The installation had two symmetrically placed plate-and-frame cross-flow modules with flat sheet membranes, which allowed obtaining duplicate test results for a given membrane. The construction of the modules is presented elsewhere [13]. The working area of each membrane was 0.0024 m^2^. The commercial membranes (UE10 and UE50) manufactured by TriSep Corporation (Goleta, CA, USA) were used for the studies. The membranes were made of PES with the addition of PVP [13]. The nominal molecular weight cut-off (MWCO) declared by the manufacturer was 10 kDa and 100 kDa for the UE10 and UE50 membranes, respectively. The UF tests were carried out using both pristine membranes and membranes that had been pre-destructed by soaking in alkaline solutions for 20 months.

Plunger pump model 3CP1221 (CAT PUMPS, Hampshire, England) pumped the feed from the tank (2 L) through the modules, achieving a feed flow rate of 1 m/s. The feed was returned to the tank immersed in the cooling bath, which allowed the feed temperature to be maintained at 293–295 K. The UF studies were performed at the transmembrane pressure (TMP) in the range of 0.05–0.3 MPa.

### 2.2. Working Solutions

The repeated cleaning of membranes with alkaline solutions can cause degradation. Therefore, the resistance of the PES membranes to such solutions was investigated. Membrane cleaning solutions were prepared from Insect and Wheel concentrates, the use of which reduced fouling during car wash wastewater separation [13]. Cleaning agents containing NaOH (pH 11.5) are applied in commercial car washes to both remove insects from cars (Insect) and wash their rims (Wheel). The composition of the cleaning agents is presented elsewhere [13,22]. Insect and Wheel concentrates contain up to 5 g/L NaOH, 8.5 g/L anionic surfactants, and 24 g/L nonionic surfactants. In the studies, the concentrates of these agents were diluted to a concentration of 0.5% *v*/*v*, which corresponds to the concentration used in a car wash. The COD value for these solutions was 1210–1250 mg O_2_/L. The membranes were also rinsed with deionized water (DI). Moreover, DI was used as a feed to determine the maximum permeate flux (J_max_) and pure water flux (J_PWF_).

Long-term degradation studies have been carried out using alternating filter wastewater and washing membranes. The detergents and hydrowaxes used in car washing cause the significant fouling of UF membranes [13]. A synthetic car wash wastewater was prepared by the addition of a mixture of surfactants (Active Green) and waxes (Hydrowax), and applied to water in commercial car washes (EuroEcol, Łódź, Poland). The turbidity of this wastewater was 6.27 NTU, the chemical oxygen demand (COD) was 3790 mg O_2_/L, and the concentration of anionic surfactants was 675 mg/L.

During the UF process, the membrane separation properties can change. To analyze this phenomenon, the 0.5 g/L solutions of dextrans with a molecular weight within the range of 70 and 500 kDa (Polfa, Łódź, Poland) were used.

### 2.3. Analytical Methods

The long-term contact of PES membranes with NaOH solution caused membrane degradation, which led to an increase in the permeate flux and a deterioration of separation properties [31]. Hence, in this work, the changes in the rejection of dextrans (molecular weight of 70–500 kDa), total organic carbon (TOC), COD, and surfactants were studied. The rejection coefficient was calculated based on Equation (1):

(1) $$R=\left(1-\frac{C_{P}}{C_{F}}\right)\;100\%$$

where C_F_ is the concentration of a component in feed, and C_P_ is the concentration of a component in permeate.

The Hach cuvette tests were used to determine the concentration of surfactants (LCK 334—nonionic, LCK 344—anionic) and COD (LCK 1014). The concentration of dextrans was analyzed using a high-performance liquid chromatograph (UlitiMate 3000, Dionex, Sunnyvale, CA, USA) with PolySep-GFC-P 4000 column (Phenomenex, Torrance, CA, USA). The TOC concentration was measured using a multi N/C 3100 analyzer (Analytik Jena, Jena, Germany).

The degradation phenomenon causes changes in the membrane surface, which were observed using an atomic force microscope (AFM, NanoScope V Multimode 8, Bruker Corp., Billerica, MA, USA) and a scanning electron microscope (SEM, SU8020, Hitachi, Tokyo, Japan). The functional groups presented onto the membrane surfaces were identified by the attenuated total reflection Fourier transform infrared spectroscopy (ATR-FTIR) using a Nicolet 380 FT-IR spectrophotometer with Smart Orbit diamond ATR accessory (Thermo Electron Corp., Austin, TX, USA). More details on the measurement methods can be found in the previous work [13]. Changes in the surface charge of the membranes were determined by the use of an electrokinetic analyzer (SurPASS, Anton Paar, Graz, Austria), using a 0.001 M KCl as the electrolyte. The measurements were carried out in the pH range of 2–8, with three repetitions for each measurement point.

## 3. Results and Discussion

### 3.1. The Influence of Cleaning Agents on the Performance PES Membranes

**Figure 2:**
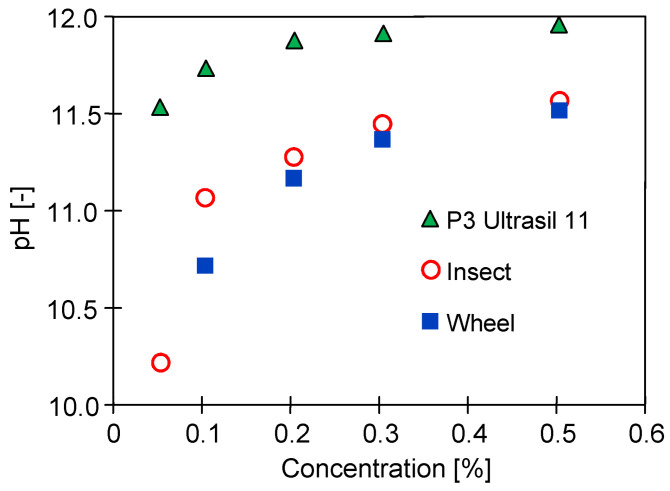
Changes in pH value depending on the concentration of P3 Ultrasil 11, Wheel, and Insect in cleaning solutions.

At a car wash, alkaline cleaning agents containing NaOH are used to remove insects from the car body and clean rims [13,22]. Dosing pumps mix the commercial concentrates of these agents with processing water and the resulting solution is applied to the surface of the car with a nozzle at a pressure of over 10 MPa. The cleaning solutions contain 0.3–0.5% *v*/*v* agent concentrate. The results shown in Figure 2 indicate that the pH of such solutions is approximately 11.5. Such values are acceptable for the PES membranes, whose manufacturers declare the ability to filter solutions with pH values as high as 12 [17,23]. The presented pH values of the tested Insect and Wheel cleaning solutions were slightly lower than the pH of the solutions of P3 Ultrasil 11, which is recommended by membrane manufacturers for the cleaning of UF membranes contaminated with oils and fats [25,35,36].

**Figure 3:**
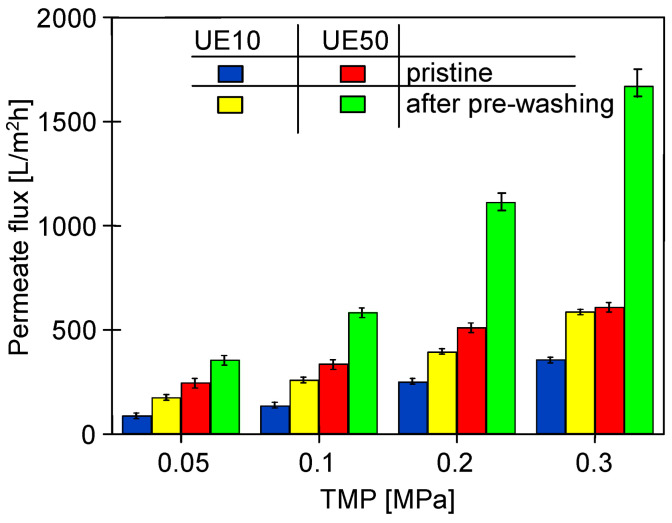
The influence of the pre-washing (2 h, TMP = 0) of pristine PES membranes with the 0.5% Insect solution on the maximum permeate flux (J_max_) measured during the UF of deionized water.

Since the commercial UE10 and UE50 PES membranes used in the studies contained preservatives, they were soaked (1 day) and then rinsed with deionized water prior to UF testing. For pristine membranes prepared in this way, the maximum permeate flux (J_max_) at TMP = 0.2 MPa was 137 L/m^2^ h for UE10 and 338 L/m^2^ h for UE50. It was previously reported that washing pristine membranes in an alkaline solution results in the more effective removal of preservative-blocking pores [31,35]. This was also confirmed in the present study (Figure 3). The application of Insect solution for washing pristine membranes (2 h, TMP = 0) resulted in an increase in J_max_ from 137 to 396 L/m^2^h for UE10, and from 338 to 1113 L/m^2^h for UE50 (TMP = 0.2 MPa). A greater increase was found for UE50 membranes (about 3.3 times) compared to UE10 (about 2.9 times), as the former membranes with a claimed MWCO of 100 kDa had larger pores than the latter (10 kDa). In addition to the removal of preservatives, the observed increase in flux can also be attributed to an increase in pore size after rinsing the PES membranes with alkaline solutions [38]. It has been shown that the contact of the polymer membrane with NaOH solution causes the loosening of the polymer chains and as a result, the skin layer becomes more permeable to water [32,35].

**Figure 4:**
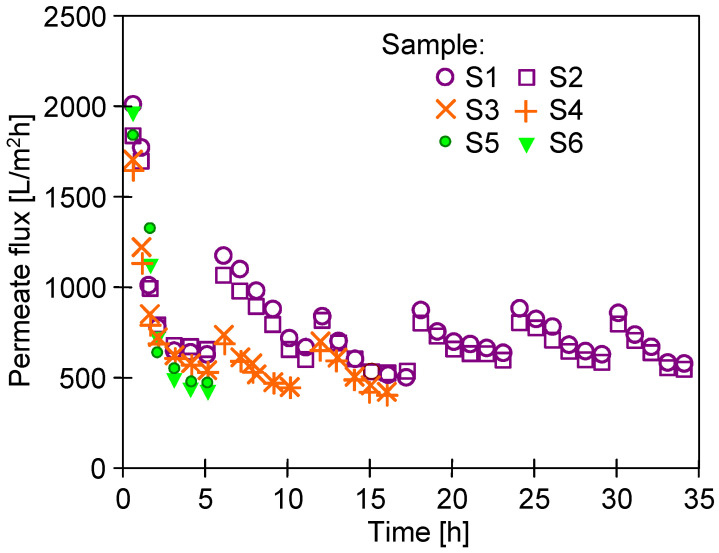
Changes in the maximum permeate flux (J_max_) during the filtration of deionized water through various samples of the UE50 membrane (compaction effect). TMP = 0.3 MPa. Samples S1–S6 were pre-washed with the 0.5% Insect solution (2 h, TMP = 0).

However, the increase in J_max_ observed after membrane pre-washing with the Insect solution was unstable. In the following hours of deionized water filtration, a systematic decrease in the permeate flux was observed. For the UE10 membranes, the permeate flux (TMP = 0.3 MPa) stabilized at the level of 320–350 L/m^2^h. In the case of the UE50 membranes, after 5–6 h of membrane compression, the flux stabilized at the level of 500–600 L/m^2^h (Figure 4). Similarly, large flux decreases in the initial period of the exploitation of PES membranes were presented in [28]. The tested UE50 membrane samples (S1–S6) were taken from various places on the 30 × 60 cm sheet. The different initial permeate flux values indicated some heterogeneity in the large membrane sheets produced. Differences in flux for commercial membranes were also reported previously [35]. However, these differences disappeared after 5–10 h of the process, and comparable permeate flux values were obtained (Figure 4).

**Figure 5:**
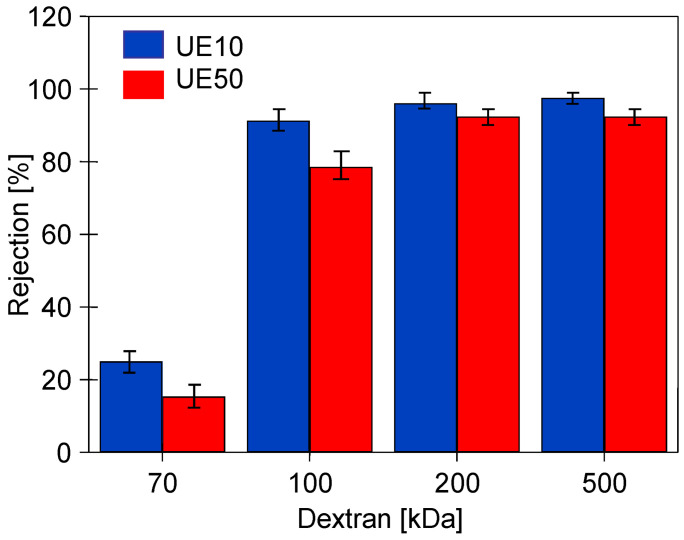
Dextran (0.5 g/L) rejection by the studied pristine PES membranes pre-washed with the 0.5% Insect solution. TMP = 0.1 MPa.

Chemical agents, by changing the structure of the membrane surface, cause not only changes in membrane permeability but also in selectivity, which was assessed in this study by examining the dextran rejection. The initial values obtained for compacted pristine membranes are presented in Figure 5. Despite a significant difference in the MWCO values declared by the manufacturers (UE10—10 kDa and UE50—100 kDa), both types of membranes showed similar separation properties. The UE50 membranes, due to their higher initial permeability, are more prone to fouling [13]. Thus, improved rejection for the UE50 membranes could be due to higher fouling intensity, as the deposit reduces the permeate flux and simultaneously improves the separation properties of the membranes [21]. The MWCO values different than those claimed by the manufacturer for PES membranes were also found in previous work [39]. During the filtration of surfactant solutions, this was explained by the concentration polarization, especially at higher fluxes [40]. Increasing the surfactant concentration in the polarization layer in the vicinity of the membrane surface can lead to monomer and micellar formation, and as a result, the PES 30 kDa membrane removed 60–80% of the anionic surfactants, similar to the PES 10 kDa [41].

**Figure 6:**
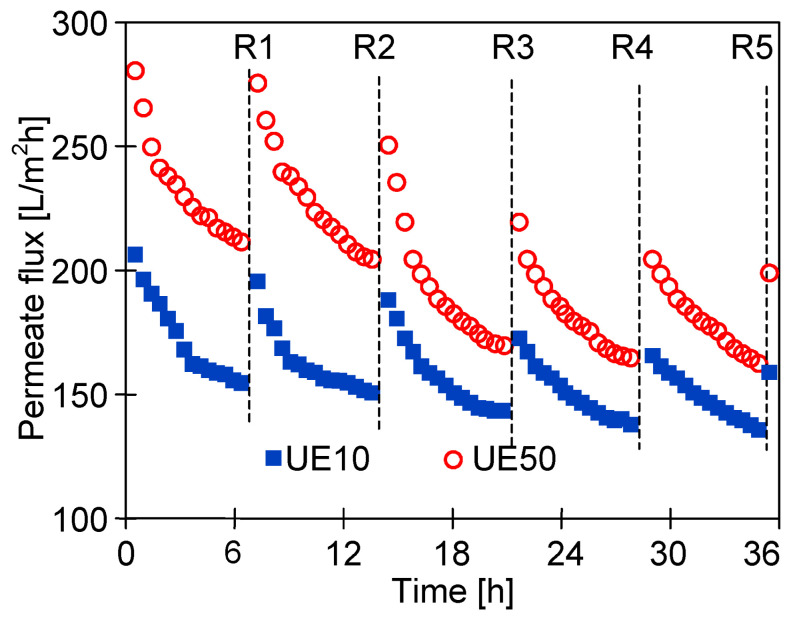
Changes in the permeate flux during the UF of the synthetic wastewater (solution containing 0.5% Active Green and 0.2% Hydrowax). TMP = 0.1 MPa. R1–R5—denote the rinsing of the membranes with deionized water (2 L). After rinsing the modules were filled with DI water and the membranes were soaked for 15 h.

Similarly to dextrans, the ingredients present in car wash wastewater also cause fouling, which affects membrane performance [13]. The results of the filtration of the synthetic car wash wastewater containing the foaming agent (Active Green) and waxes forming a protective layer on the car surface (Hydrowax) are presented in Figure 6. The experiment was realized in five cycles of wastewater filtration. After each cycle, the membranes were rinsed with deionized water (2 L) by circulating water in the membrane installation without any filtration, and then the membrane modules were filled with water, and the membranes were soaked for 15 h.

**Figure 7:**
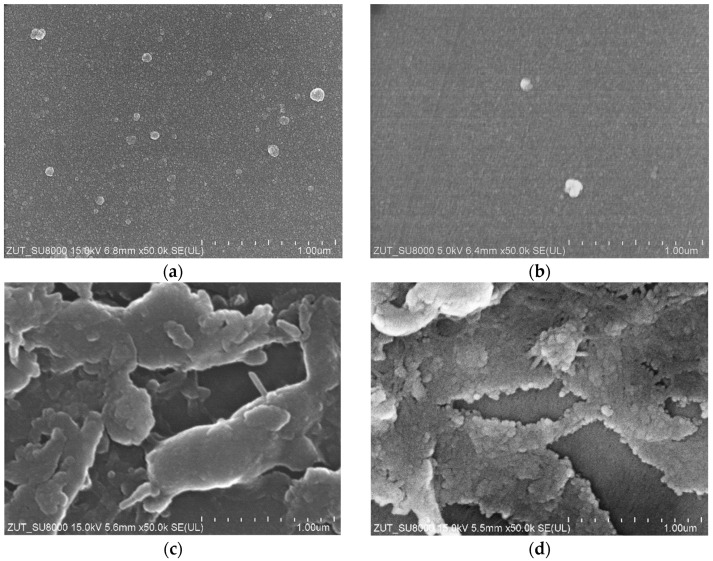
SEM images of the membrane surfaces. Pristine membranes: (**a**) UE10, (**b**) UE50, and covered by deposit: (**c**) UE10, (**d**) UE50. The fouled membrane samples taken after the completion of the UF tests are presented in Figure 6.

It was found that for the tested wastewater, the permeate flux was twice lower than that obtained for deionized water (Figure 4). The results obtained during the UF of the synthetic wastewater showed a systematic decrease in the permeate flux in subsequent cycles; hence, rinsing with DI water was not effective enough in cleaning the membranes. As a result, after 36 h of UF, the permeate flux decreased from 200 to 160 L/m^2^h (UE10) and from 280 to 200 L/m^2^h (UE50). Similar flux changes caused by fouling were shown for PES membranes in [24,25]. It was indicated that the main reason for the flux deterioration was the formation of filter cake, but a significant contribution of internal fouling was also found. Contaminants accumulating inside the pores are difficult to remove, and as a result, despite the repeated rinsing of the membranes (Figure 6, R1–R5), a progressive decrease in the permeate flux is observed. The formation of a deposit on the surface of the membranes was also confirmed by the SEM observations (Figure 7). The collected images were similar for both types of the membranes tested (UE10 and UE50). Despite rinsing with water, most of the membrane surface was covered with deposits. However, it created a porous structure, thus the flow through the membrane was still possible.

**Figure 8:**
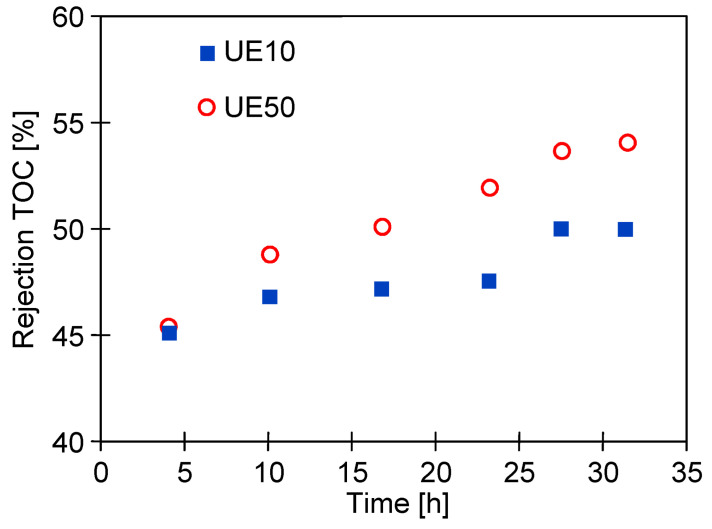
Changes in the rejection of TOC during the UF of the synthetic wastewater containing 0.5% Active Green + 0.2% Hydrowax. TMP = 0.1 MPa. The results correspond to the data presented in Figure 6.

The formation of deposits on the membrane surface usually improves the membrane’s separation properties [21]. This was confirmed by the results of the TOC analysis, which showed that during wastewater filtration (Figure 6) the retention of organic compounds increased with the process time (Figure 8). In the tested case, the flux decreased by 20% for the UE10 membranes, and by 29% for UE50, which indicates that fouling was greater in the former case. As a result, a slightly higher TOC rejection was obtained for the UE50 membrane.

### 3.2. Long-Term UF with Periodical Alkaline Rinsing

**Figure 9:**
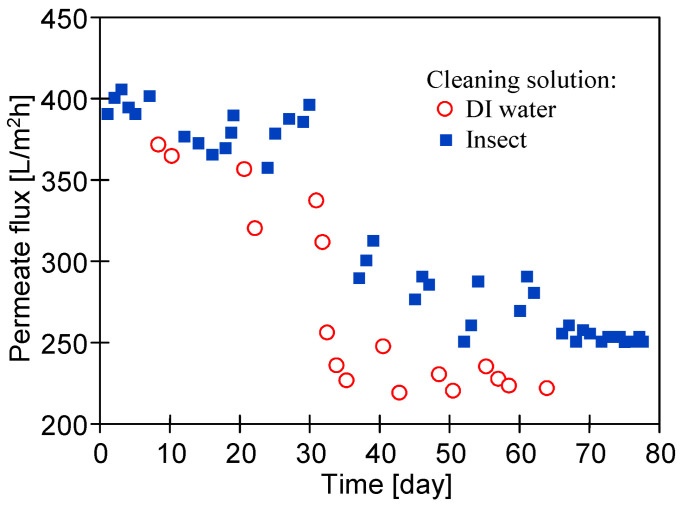
Changes in pure water flux (J_PWF_) after filtration of synthetic wastewater with periodical membrane cleaning (1 h, TMP = 0) using: DI water only (red circle) or 0.5% Insect solutions (blue squares represent J_PWF_ measured after membrane washing with alkaline agent; feed: DI water). TMP = 0.1 Mpa. Membrane: UE50.

The low effectiveness of rinsing the membranes with DI water was also shown in previous work, in which a stable permeate flux during the UF of car wash wastewater was obtained by periodically washing the membranes with Insect solution [13]. To determine how the repetition of this procedure affects the performance of the PES membranes, the UF process of the synthetic car wash wastewater with cyclic washing was carried out for 3 months. After each washing, the maximum permeate flux was determined using deionized water as a feed. The obtained values are presented in Figure 9. Wastewater filtration was carried out 5–6 h/day, after which the membranes were washed with the 0.5% Insect solution (1 h) by the circulation of the cleaning solution in the UF installation (no filtration was performed).

The obtained results show the importance of the systematic repetition of this operation. The simple rinsing of the fouled membrane with DI water only (Figure 9, red circle) was not efficient in the removal of the fouling layer, and as a result, during a longer time of operation, the permeate flux could not be restored even after alkaline cleaning (Insect). This phenomenon was intensifying with the duration of the process. For example, after 8 days of the filtration of the synthetic wastewater when only alkaline cleaning was applied, the initial permeate flux was successfully restored (approximately 400 L/m^2^h). The DI water rinsing applied subsequently (2 days) was not efficient in the removal of the fouling layer and as a result, the permeate flux was lower (approximately 375 L/m^2^h after 11 days). However, the reapplication of alkaline cleaning resulted in the restoration of the permeate flux up to 400 L/m^2^h. Such an approach was efficient during the first 30 days of the study. Later, this effect was smaller. The largest drop in the permeate flux (J_PWF_) after rinsing with DI water only was observed after 30 days, when no alkaline cleaning was performed for 5 days. After 70 days of the UF process, the pure water flux stabilized at the level of 250 L/m^2^h (Figure 9).

**Figure 10:**
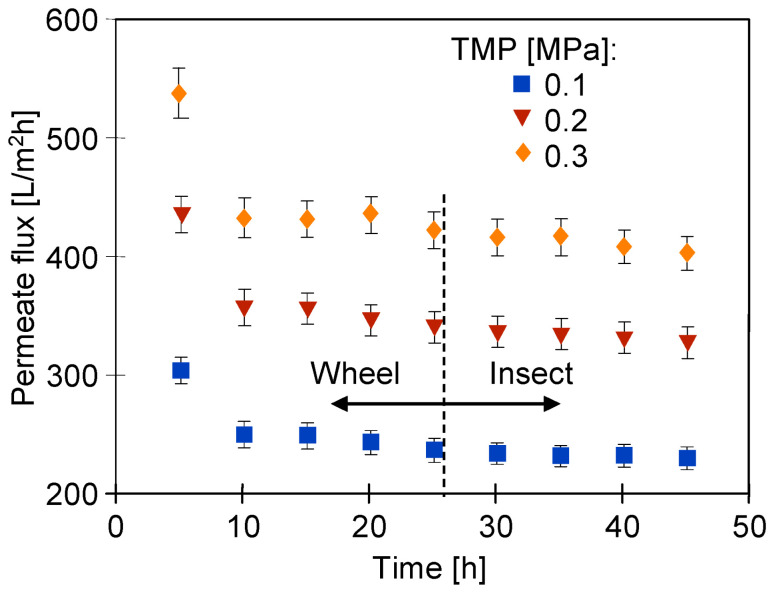
Changes in the pure water flux after 25 h of rinsing with the 0.5% Wheel solution and 0.5% Insect (20 h). The UE50 membranes after the UF of the synthetic wastewater are presented in Figure 9.

After 78 days of UF tests (Figure 9), the membranes were washed several times using 0.5% solutions of Wheel (25 h) and Insect (20 h). During this procedure, the membranes in the modules were washed for 5 h/day, then the alkaline solution was replaced with DI water and the pure water flux was determined (Figure 10). Despite long-term contact with the alkaline solution, no such significant increase in the flux was observed for the pristine membranes (Figure 3). However, the membrane performance stabilized at a level appropriate for a given TMP value.

**Figure 11:**
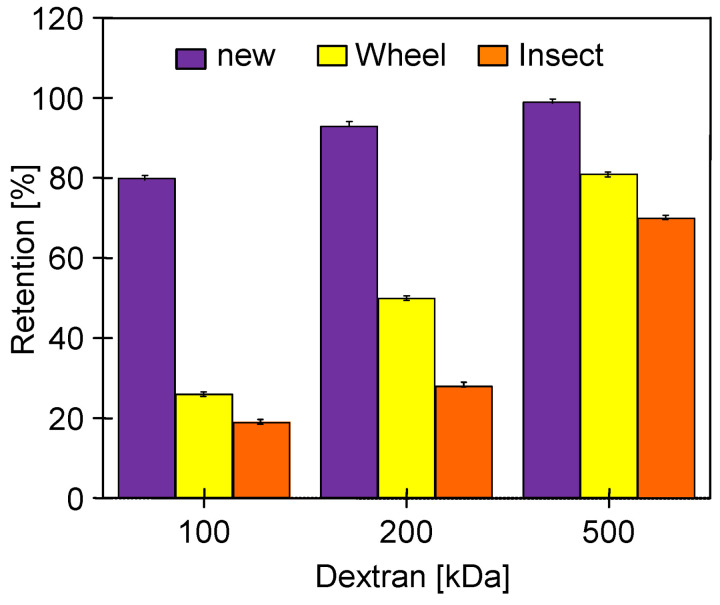
Dextran rejection by the UE50 membranes applied during the treatment of the synthetic car wash wastewater. Wheel—membranes after 78 days of the UF process (Figure 9) rinsed for 25 h with the 0.5% Wheel solution. Insect—membranes rinsed for another 20 h with the 0.5% Insect solution.

After 25 h of using the Wheel solution, it was replaced with an Insect solution, which, however, did not significantly change the values of the pure water flux (Figure 10). Although the change in cleaning solutions had no effect on J_PWF_, the dextran separation studies showed that the additional 20 h of washing with Insect solution resulted in a decrease in retention (Figure 11). The pristine membranes rejected over 93% of the 200 kDa dextran and almost 100% of the 500 kDa dextran. After 78 days of the UF process (Figure 9) and 25 h of washing with the 0.5% Wheel solution, the 500 kDa separation decreased to 82%, and after another 20 h of washing with the 0.5% Insect solution—to 71%. The retention coefficient of 100 and 200 kDa dextrans decreased by more than two-fold. This result indicates that the application of alkaline agents resulted in an increase in the pore size of the tested membranes. A significant reduction in the dextran rejection was also achieved during static tests in which the membranes were soaked in Insect and Wheel solutions for 18 months [22].

**Figure 12:**
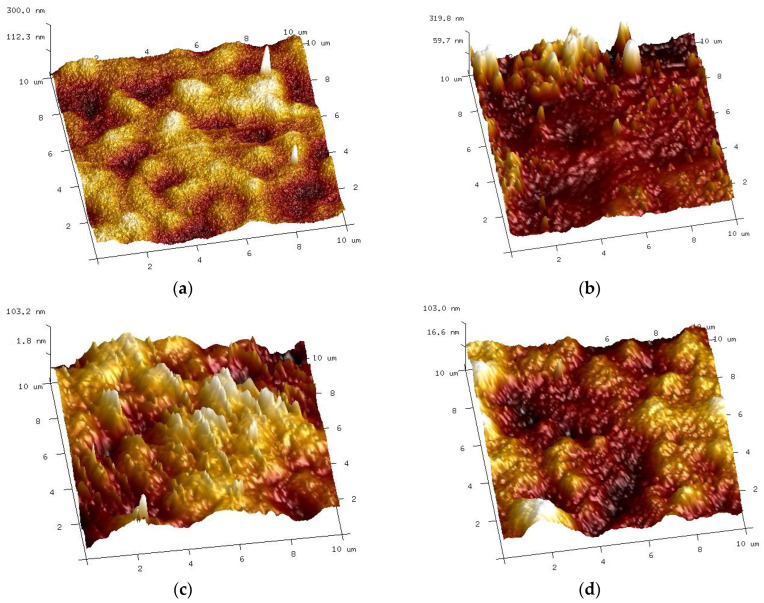
AFM images of UE50 membranes surface: (**a**) pristine and (**b**) washed with 0.5% Wheel solution and 0.5% Insect solution (Figure 10) after 78 h of UF process (Figure 9), and after 20 months of soaking in (**c**) 0.5% Insect solution and (**d**) 0.5% Wheel solution.

AFM analysis confirmed that the membrane surface changed during cyclic membrane washing with the 0.5% Wheel and 0.5% Insect solutions (Figure 12b). Despite intensive washing with alkaline agents (Figure 10), small amounts of contaminants still remained on the membrane surface in some places. As a result, the values of the surface roughness parameters increased (Table 1, washed).

Similarly, there were significant changes in the surface structure of the membranes after 20 months of soaking, although these were dependent on the type of alkaline agent used (Figure 12c,d). Compared to the pristine membranes (R_q_ = 21.5 nm), the surface of the membrane soaked in the 0.5% Insect solution became rougher (R_q_ = 27.9 nm), contrary to the sample from the 0.5% Wheel solution, which was slightly smoother (R_q_ = 20.3 nm) (Table 1, soaked). The obtained result indicates that despite the similar NaOH content, even slight differences in the composition of the remaining cleaning compounds [22] may affect the degradation of the PES membranes by the alkaline agents used.

### 3.3. Filtration of Alkaline Solutions

**Figure 13:**
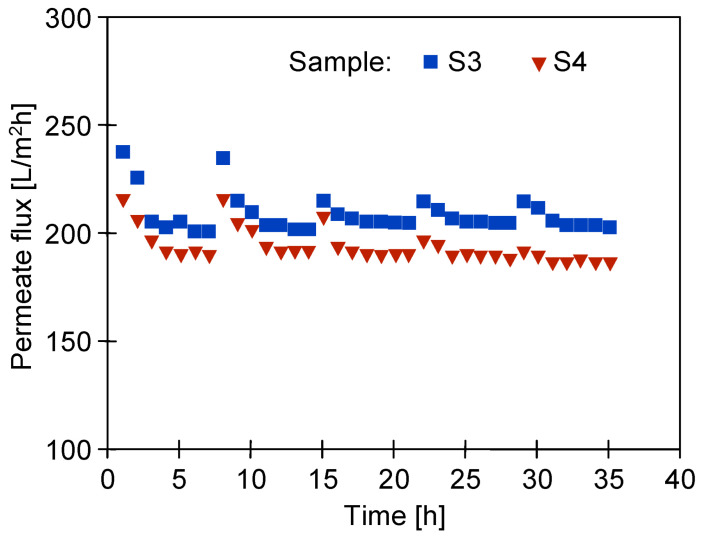
Changes in the permeate flux during the filtration of the 0.5% Insect solution (pH = 11.5) through the pristine UE50 membranes (TMP = 0.1 Mpa). The process was conducted for 6–7 h per day, after which the installation was rinsed and filled with DI water overnight.

The fouling of the membranes caused by wastewater separation (Figure 7c,d) not only reduces membrane permeability, but the deposit can also protect polymer surfaces from the harmful action of NaOH [41]. Hence, in order to investigate the influence of alkaline agents on the destruction of the PES membrane, a 0.5% Insect solution was filtered through it for several weeks. It was already reported that contact with alkaline solutions causes the loosening and degradation of polymer chains [30]. Previous static tests showed that soaking the membranes in Insect and Wheel solutions for several months resulted in the removal of PVP from the membranes and the cracking of the PES chains [22]. Unlike static conditions, during the UF process in cross-flow modules, the generated shear forces can accelerate the removal of components such as PVP from the membrane matrix [17]. For this reason, in order to investigate the mechanism of the PES membrane degradation, in addition to washing the membrane surface (without any filtration), the experiments were conducted during which an alkaline solution flowed through their pores. Compressed pristine UE50 membrane samples were used for the tests (Figure 4, samples S3 and S4), through which the 0.5% Insect solution was filtered (TMP = 0.1 MPa) for 8 weeks. The UF process was carried out for 6–7 h per day, after which the installation was rinsed and filled with deionized water overnight. The process conducted in this way allowed the permeate flux to be stabilized after just 2 days of testing (Figure 13, from 15 h). After the night break, slightly higher flux values were obtained, which resulted from the decompression of the membranes during standstill and osmotic rinsing by deionized water. In the first week of the 0.5% Insect solution filtration, the permeate flux stabilized at around 200 L/m^2^h (sample S3) and 180 L/m^2^h (sample S4). In the following weeks, a slight increase in the permeate flux was observed.

**Figure 14:**
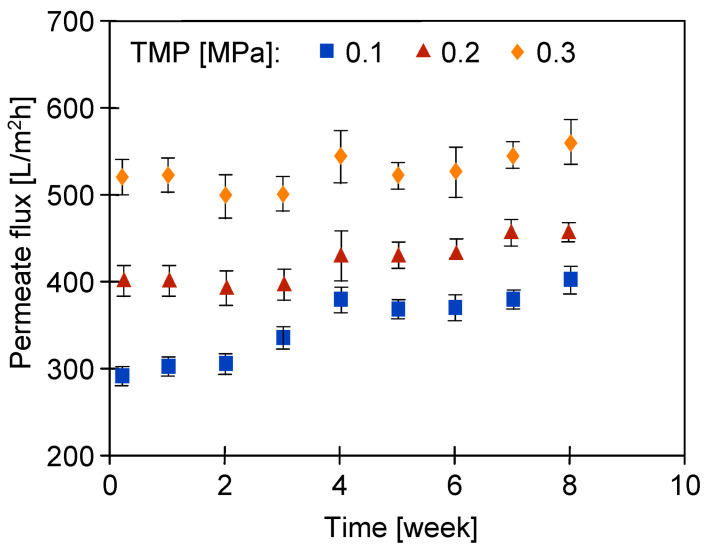
Changes in the maximum permeate flux (feed—DI water) during 8 weeks of the filtration of the 0.5% Insect solution. The process was conducted for 6–7 h per day, after which the installation was rinsed and filled with DI water overnight. Subsequently, J_max_ was measured using DI water.

Sample S4 showed 10% lower permeability, but the nature of the permeate flux changes for both the UE50 membrane samples in the examined period was similar. The average J_max_ values of the permeate flux obtained for them are presented in Figure 14. Over time, there was a slight increase in its value, which indicated an increase in the pore sizes in the separation layer. Smaller changes were observed when TMP was increased to 0.3 Mpa, which indicated that the matrix of the membranes, which was loosened due to the influence of the alkaline agent, was tightened due to compression at a higher TMP value.

**Figure 15:**
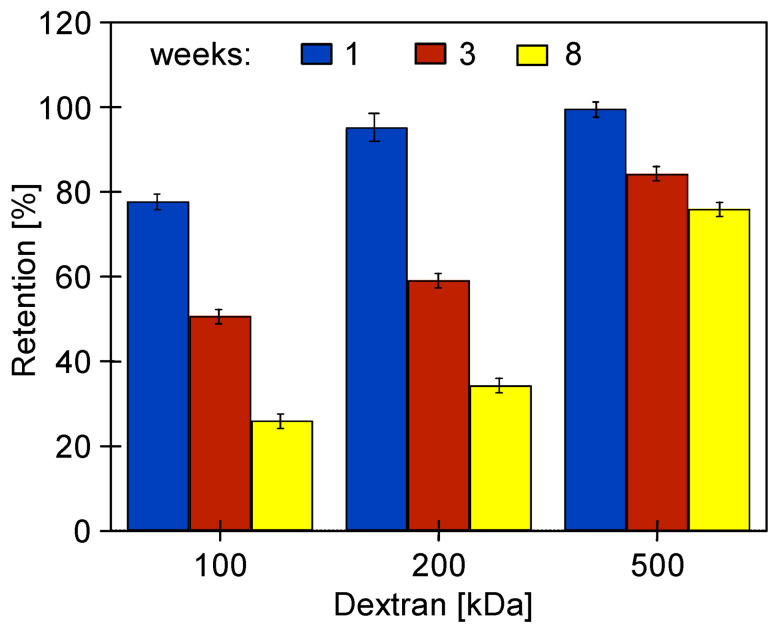
The influence of the time (weeks) of the filtration of the 0.5% Insect solution (pH = 11.5) on dextran rejection by the UE50 membrane. TMP = 0.1 Mpa.

The increase in the permeability of the PES membranes due to the influence of the long-term filtration of the 0.5% Insect solution was confirmed by a decreased retention of dextrans (Figure 15). These changes increased with the time of the process. After 8 weeks, particularly large decreases in retention to the level of 20–30% were observed for dextran 100 and 200 kDa. After this time, the rejection of dextran 500 kDa decreased from 97 to 75%. A significant increase in water permeability and a decrease in the retention of dextran as a result of washing membranes with NaOH solution was also presented in [30]. In static tests, after 18 months of soaking in the Insect solution, the UE50 membranes retained dextrans more than twice as badly [22]. This result indicates that during cross-flow operation, the alkaline cleaning agents can cause membrane degradation to a much greater extent than that occurring during the long-term soaking of the membranes in these solutions.

**Figure 16:**
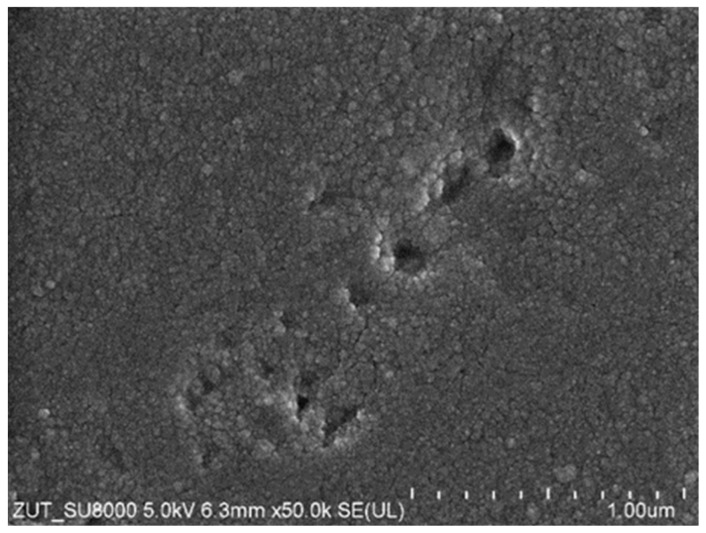
SEM image of the surface of the UE50 membrane after 8 weeks of the filtration of the 0.5% Insect solution.

The SEM observations showed that the decrease in the dextran rejection resulted not only from the loosening of the structure and the enlargement of the pores in the skin layer but also from its degradation resulting in damage on a micro-scale. On the surface of the membranes, in a few places, holes with sizes up to 0.3 μm were observed (Figure 16). The creation of the channels of such sizes allowed the feed to flow, which resulted in an increase in the dextran content in the permeate and, as a result, a reduction in its rejection (Figure 15).

**Figure 17:**
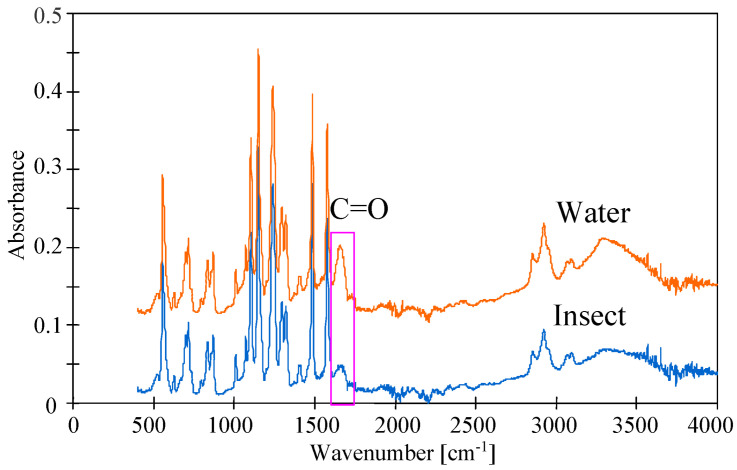
ATR-FTIR spectra of UE50 PES membranes after DI water filtration (water) and after 8 weeks of 0.5% Insect solution filtration (Insect).

PES is relatively hydrophobic, which may accelerate the membrane fouling phenomenon [38]. To limit this, PVP is added to the PES membranes, which increases their hydrophilicity. However, the presence of PVP reduces the resistance of the membranes to NaOH solutions [29,31]. It was confirmed that the UE10 and UE50 membranes contain PVP [13,22]. ATR-FTIR studies (Figure 17) showed that after 8 weeks of the filtration of the 0.5% Insect solution, the intensity of C=O amide bound at 1679 cm^−1^, which is characteristic for PVP [19,42], decreased significantly. The intensity of the remaining peaks representing PES practically did not change. This result confirms that the observed decrease in dextran rejection (Figure 15) and the changes in the structure of the membrane surface (Figure 16) may result from the leaching of PVP from the membrane matrix [22]. The above confirms the opinion that there are some drawbacks such as the inevitable leaching of the blended hydrophilic materials from the polymeric membranes after long-term use [15].

### 3.4. Long-Term Tests of Membrane Aging

The filtration of the 0.5% Insect solution carried out for 8 weeks showed significant changes in the structure and composition of the PES membranes (Figure 16 and Figure 17). However, from the application perspective, an 8-week period is relatively short, since it is expected that the membranes would be operating for months or years. Therefore, in order to determine how a longer period of contact with alkaline agents will contribute to the degradation of the PES membranes, the UE50 membranes soaked for 20 months in the 0.5% Insect and 0.5% Wheel solutions were used for subsequent tests. After this period, a significant degradation of the membrane surface was observed (Figure 12).

**Figure 18:**
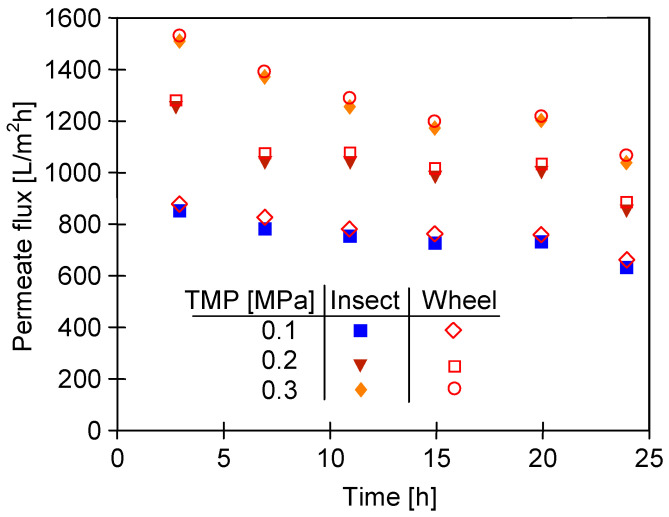
Changes in the maximum permeate flux (feed—DI water) during the filtration of the 0.5% Insect solution through the UE50 membranes, previously soaked for 20 months in Insect and Wheel solutions (pH = 11.5). The process was conducted for 5–6 h per day, after which the installation was rinsed with DI water. Subsequently, J_max_ was measured using DI water.

The soaked membrane samples were assembled in two UF modules operated in parallel and the filtration of the 0.5% Insect solution (TMP = 0.1 Mpa) was carried out for 24 days. After 5–6 h of filtration, the modules were rinsed with deionized water and the maximum permeate flux was determined. The results obtained for both types of membranes are shown in Figure 18. The flux values were twice those obtained for the pristine membranes (Figure 14). This shows that soaking the membranes for 20 months led to a significant increase in their pore size. Despite differences in surface topography (Figure 12c,d), the permeate flux for the membranes soaked in the 0.5% Wheel solution was only 10–30 L/m^2^h higher than that obtained for the membranes soaked in the 0.5% Insect solution. The fluxes decreased with the process time, which could be due to the tightening of the separation layer, which was previously loosened during the 20-month soaking period.

**Figure 19:**
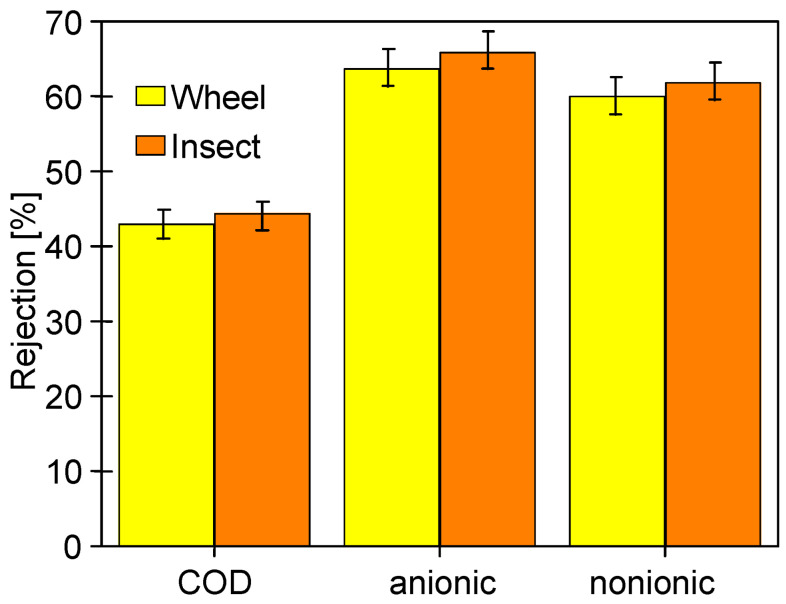
Rejection of COD and surfactants (anionic and nonionic) during UF of 0.5% Insect solution through UE50 membranes soaked for 20 months in alkaline solutions. TMP = 0.1 Mpa.

**Figure 20:**
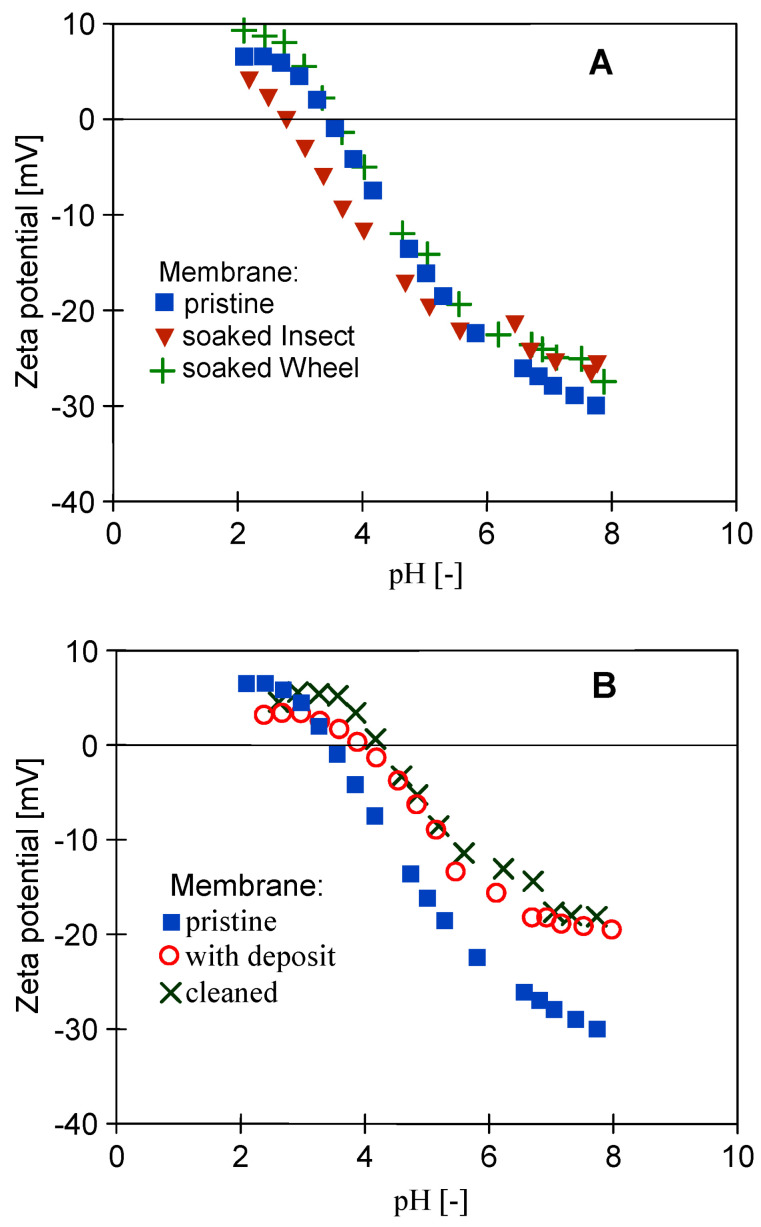
Comparison of the zeta potential of the surface of the pristine UE50 membrane: (**A**) soaked in the 0.5% Wheel solution and 0.5% Insect solution and (**B**) fouled during the UF of the synthetic wastewater (with deposit) and then cleaned.

In order to analyze the influence of the long-term soaking of the membranes on their separation properties, the rejection of COD and surfactants present in the 0.5% Insect solution was determined (Figure 19). The membranes soaked in the 0.5% Wheel solution showed slightly higher permeability, so as expected, slightly lower retention (by 2–3%) was recorded for these membranes compared to those soaked in the 0.5% Insect solution. The rejection of the anionic surfactant was higher than that of the nonionic surfactant, both by the membranes soaked in Insect and Wheel solutions. Zeta potential measurements carried out after the UF tests showed that long-term contact with alkaline agents does not significantly change the electronegativity of the membrane surfaces (Figure 20). Due to the dissociation of the anionic surfactants, they were more strongly repulsed by the membranes, which increased their rejection. Similar conclusions for PES membranes based on Zeta potential changes were presented in other works [43,44].

**Figure 21:**
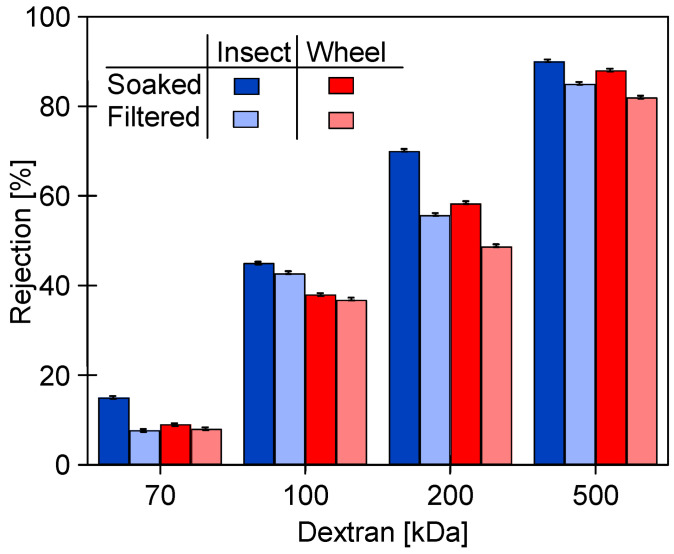
Dextran rejection by UE50 membranes soaked for 20 months in 0.5% Insect and 0.5% Wheel solutions, and then applied in 0.5% Insect solution filtration (24 h, Figure 18).

After 24 h of the filtration of the 0.5% Insect solution (Figure 18), the membranes were rinsed with deionized water, and dextran rejection was analyzed (Figure 21). The obtained results confirmed that the effects of static and dynamic aging tests differ significantly. The soaking of the pristine membranes for 20 months in the alkaline agents deteriorated their separation properties. For example, in the case of the 0.5% Insect solution, the rejection of 500 kDa dextran decreased from 96% to 92%, and for 100 kDa from 80% to 46%. For the membranes soaked in the 0.5% Wheel solution, the rejection of these dextrans decreased to 90% and 39%, respectively. However, the deterioration of separation properties was significantly lower than that observed for the membranes after 8 weeks of the filtration of the 0.5% Insect solution (Figure 15). It follows that the application of the simple soaking of the membranes to determine their resistance to alkaline agents [20,21] does not show the entire changes in their structure that can be caused by cleaning solutions during their filtration in UF modules. This is confirmed by the results of the impact of the filtration of the 0.5% Insect solution carried out for 24 h, for which, despite the short contact time, dextran rejection decreased by another few percent (Figure 21, filtered) compared to the values observed after soaking (Figure 21, soaked).

### 3.5. Treatment of Synthetic Car wash Wastewater

**Figure 22:**
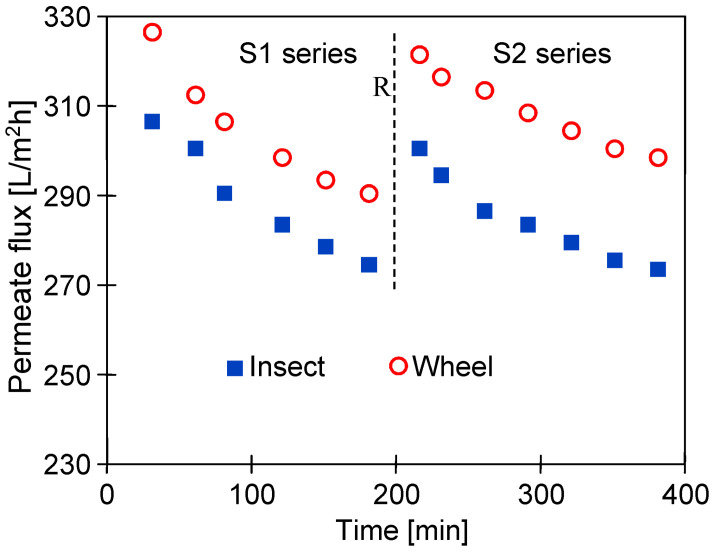
Changes in the permeate flux during the UF of the synthetic wastewater (0.5% Turbo Green + 0.2% Hydrowax solution). Initially, the UE50 membranes were soaked for 20 months in the 0.5% Insect (Insect) and 0.5% Wheel solutions (Wheel). R—denotes the cleaning of membranes with the 0.5% Insect solution (2 h). TMP = 0.1 Mpa.

**Figure 23:**
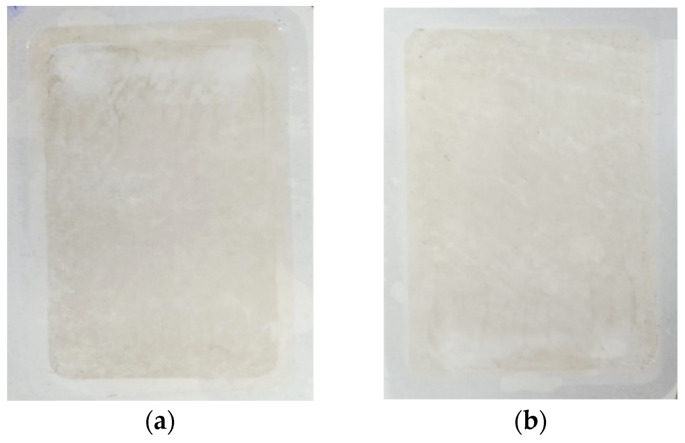
Images of the surface of the UE50 membranes collected from the UF modules after the treatment of the synthetic wastewater. Initially, the membranes were soaked for 20 months in (**a**) the 0.5% Wheel solution and (**b**) the 0.5% Insect solution.

In the last part of the research, the influence of the changes in membrane properties presented in Section 3.4 on their performance during the filtration of synthetic wastewater (0.5% Turbo Green + 0.2% Hydrowax mixture in water) was investigated. Two series of wastewater filtration were carried out. After completing series S1, the membranes were washed with the 0.5% Insect solution (2 h), which allowed the initial performance to be regained. It was found that the permeate flux decreased during the UF process and depended on the solution in which the membranes were soaked (Figure 22). A permeate flux higher by 20–23 L/m^2^h was obtained for the membranes soaked in the 0.5% Wheel solution. This indicates that this agent caused a greater degradation of the membrane structure than Insect, which is also confirmed by the differences in dextran separation (Figure 21). A small amount of deposits visible on the membrane surface after the completion of the S2 series (Figure 23) confirmed that fouling was the reason for the observed decrease in the permeate flux.

**Figure 24:**
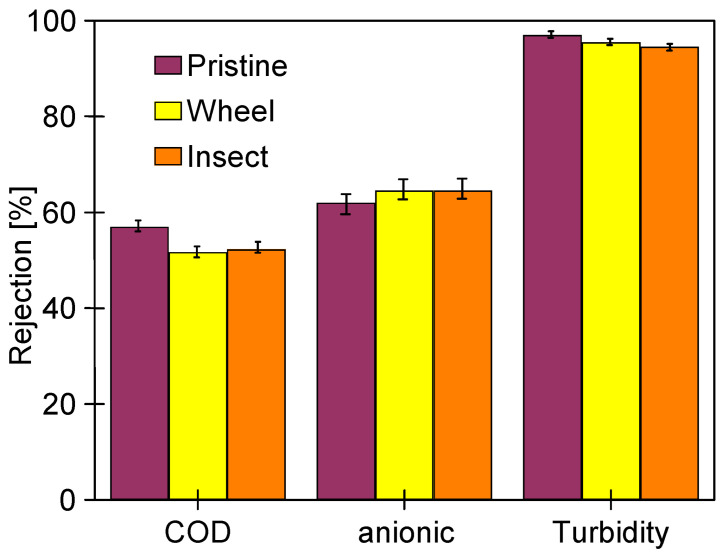
Removal of COD, anionic surfactants, and turbidity during UF of synthetic wastewater. Membrane: UE50 membrane after 24 h filtration of 0.5% Insect solution (Figure 19), previously soaked for 20 months in alkaline solutions. TMP = 0.1 MPa.

During the S1 series, after 2 h of the process (Figure 22), the samples were taken to assess the rejection of the components present in the synthetic wastewater. It was found that despite a significant deterioration of dextran rejection (Figure 21), the retention of COD, anionic surfactants, and turbidity was similar to that observed for pristine membranes. The turbidity removal was close to 95% for all membranes. The COD rejection decreased from 60 to 52%; however, it was less severe than that observed for the filtration of the 0.5% Insect solution (approximately 45%, Figure 19). Similarly efficient separation was obtained in the coagulation–filtration process, for which the turbidity and COD removal reached approximately 90% and 60%, respectively [5]. In the UF process, apart from the influence of pore sizes, the separation is also affected by the formation of a fouling layer, whose presence on the membranes’ surface was confirmed (Figure 23). This conclusion is supported by similar results obtained for the membranes soaked in the 0.5% Insect and 0.5% Wheel solutions (Figure 24). On the opposite, during filtration of the 0.5% Insect solution, which did not form deposits, lower rejection was obtained for membranes soaked in 0.5% Wheel (Figure 19). The presence of the fouling layer contributed also to the observed improved retention of the anionic surfactants compared to the pristine membrane (Figure 24).

**Figure 25:**
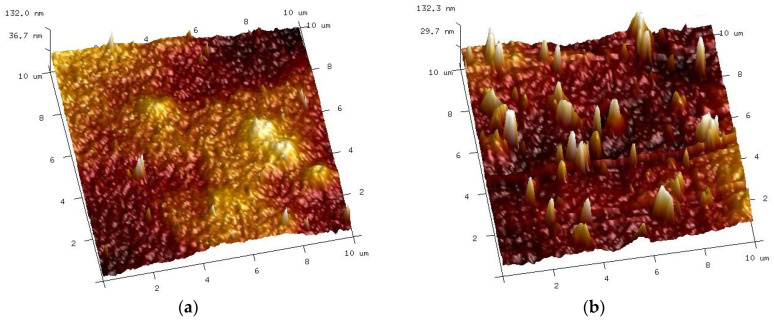
AFM images of the surface of membranes after the UF of the synthetic wastewater and washing off the precipitate are shown in Figure 23. UE50 membrane soaked in (**a**) Insect solution and (**b**) Wheel solution.

After completing the experiments presented in Figure 22, the membranes were soaked in deionized water overnight, and then the sediment visible in Figure 23 was rinsed with a strong stream of water, which allowed the white color of the membranes to be regained. The AFM images of the surface of such washed membranes are shown in Figure 25. It can be seen that the surface of the membranes applied during the treatment of the synthetic wastewater differed compared to the membranes only soaked for 20 months (Figure 12c,d). The roughness parameters for the membrane soaked in the 0.5% Insect solution decreased significantly after the UF of wastewater (Table 1, filtered), e.g., for R_q_ from 27.9 nm to 15.8 nm. On the surface of the membranes soaked in the 0.5% Wheel solution and applied in the treatment of the wastewater, the residues of sediment are visible, the presence of which increased the value of the R_q_ from 20.3 to 25.6 nm.

## 4. Conclusions

Rinsing the pristine PES membranes with water and alkaline cleaning solutions caused a significant increase in the permeate flux. The obtained result confirms that before UF studies, the commercial UF membranes should be subjected to an initial stabilization process of several hours.

The results confirmed that PVP added to PES membranes is removed from the membrane matrix during long-term contact with alkaline cleaning solutions. This causes the separation layer to loosen, which results in a more than two-fold reduction in dextran rejection.

Membrane exposure to alkaline solutions under static and dynamic conditions was found to have different effects on membrane degradation. The cross-flow filtration of a solution containing surfactants and NaOH (pH = 11.5) conducted for 8 weeks caused significantly greater changes in the structure of the membranes than those observed after 20 months of soaking the membrane in these solutions. For this reason, it can be expected that during the operation of the UF installation, the solutions used for cleaning the membranes will cause a greater degradation of the membranes than was found using the membrane soaking method.

Despite significant changes in the properties of the UF membranes (mainly the loosening of the structure of the skin layer observed in dextran rejection), the efficiency of the treatment of the car wash wastewater did not deteriorate. This indicates that not only the pore size but also the formation of a fouling layer on the membrane surface has a significant impact on the separation effect.

## Figures and Tables

**Table 1:** The parameters of the membrane surface roughness calculated based on the AFM analysis.

Sample	R_q_ [nm]	R_a_ [nm]	R_max_ [nm]
Pristine	21.5 ± 2.5	17.1 ± 3.6	179 ± 31.5
Washed	54.3 ± 18.8	39.4 ± 12.5	380 ± 93.8
Insect			
Soaked	27.9 ± 7.5	22.2 ± 3.2	295 ± 45.5
Filtered	15.8 ± 5.4	12.1 ± 2.2	230 ± 36.3
Wheel			
Soaked	20.3 ± 2.6	15.9 ± 1.8	203 ± 27.6
Filtered	25.6 ± 5.4	16.7 ± 3.2	320 ± 61.1

## Data Availability

The original contributions presented in the study are included in the article, further inquiries can be directed to the corresponding author.
